# Utilisation of indigenous knowledge to control gastrointestinal nematodes in Southern Africa

**DOI:** 10.1007/s11250-024-03941-z

**Published:** 2024-03-12

**Authors:** ET Kamba, M Chimonyo

**Affiliations:** Faculty of Science, Engineering and Agriculture, University of Venda, University Road, Thohoyandou, 0950 South Africa

**Keywords:** Water-stress, Predisposing factors, Pregnancy status, Coat colour, Sex

## Abstract

Gastrointestinal nematodes (GIN) exacerbate the impact of droughts on the survival of cattle. The inadequacies of the conventional system make it increasingly important to explore indigenous knowledge (IK) to create drought-tolerant and GIN resilient herds. The objective of the study was to assess the indigenous strategies for controlling GIN during droughts. Face-to-face interviews with experts on IK were conducted to give insight into the importance, methods and ranking of GIN control. The experts identified 86 cattle that were used to test their assertions. The control methods used were identifying cattle that were susceptible to high GIN loads using predisposing factors, diagnosis of GIN burdens using faecal appearance, and treatment using phytotherapy. Experts ranked predisposing factors as the most critical control strategy and identified body condition, class, sex, coat colour, pregnancy status and lactation status as predisposing factors to high GIN burdens. Thin, older, dark-coloured cattle, as well as pregnant and lactating cows, were considered susceptible to GIN. However, pregnancy status, coat colour and sex were significantly associated with high GIN burdens. Cows were 2.6 times more likely to have high GIN burdens than bulls. Dark-coloured cattle were 3.5 times more likely to have high GIN burdens than light-coloured ones, and the likelihood of pregnant cows was 4.9 times higher than non-pregnant cows. A dark-coloured pregnant cow was extremely susceptible to high GIN burdens. In conclusion, knowledge of predisposing factors informs selection decisions when purchasing foundation stock. Cattle that are susceptible to high GIN loads are prioritised during droughts or culled where resources are scarce.

## Introduction

Gastrointestinal nematodes (GIN) aggravate the impacts of droughts in cattle under the veld-based production systems (Dzavo et al., 2019). They utilise water in the host’s body thereby increasing the water requirements of cattle in an already water-scarce environment. Low body condition, heat stress and physical strain when travelling long distances to access feed and water weaken cattle’s immunity (Ayanlade and Ojebisi, 2019). Cattle also utilise short grasses and congregate on the few available water sources where the eggs and infective larval stages survive, exposing them to new infections (Tikyaa et al., 2019). These conditions increase the incidences and impacts of GIN infestations resulting in increased stress and mortality. During droughts, cattle with heavy GIN burdens succumb to droughts faster (Dzavo et al., 2019). Gastroenteritis increases water requirements in cattle due to water loss through diarrhoea (Vercruysse et al., 2018). Gastrointestinal nematode infestations, therefore, decrease the quality of cattle products, food security and sustainable livelihoods of households. Reducing the spread and progression of GIN to levels that negatively impact cattle health and welfare is crucial. Droughts exacerbates the impact of GIN on cattle (Marufu et al., 2011; Pfukenyi and Mukaratirwa, 2013).

The most common practice of GIN control is treatment using anthelmintics. The conventional system of GIN control mainly focuses on diagnosis and treatment. The incorrect application of anthelmintics increases the prevalence of anthelmintic resistance (Tsotetsi et al., 2013; Kelleher et al., 2020). Resource-limited communities lack the financial means to acquire anthelmintics, resulting in an inconsistent supply of drugs (Charlier et al., 2015; Garforth, 2015; Ndlela et al., 2021). Invoking indigenous knowledge (IK) in developing appropriate and sustainable interventions that can easily be adopted is important. Indigenous knowledge has more opportunities, apart from treatment methods, that can be exploited to improve cattle health under drought conditions. Although there is a plethora of literature on the use of ethnoveterinary medicine in GIN control, there is limited knowledge of comprehensive IK strategies in ensuring cattle health. The adoption of IK on maintaining animal health is poorly understood, yet its adoption broadens the opportunities for veterinary and extension services opportunities to promote these cost-effective methods. Integration of IK strategies into mainstream livestock health management can improve the adoptability of intervention programmes in grassroot communities. Farmers can benefit from the IK methods since they are locally available, environmentally-friendly and biodegradable. These attributes promote sustainability of both the environment and livelihoods of resource-limited communities. The objective of the study was, therefore, to assess the indigenous strategies for controlling gastrointestinal nematodes in cows during droughts. It was hypothesised that IK is used to control gastrointestinal nematodes of cows during droughts.

## Materials and methods

### Description of the study site

The study was conducted in Musina Local Municipality (MLM) under Vhembe District Municipality, Limpopo. The municipality is situated in the northern part of Limpopo under the coordinates 22° 20 ′ 17″S 30° 02′ 30″E / 22 33,806°S 30 04167°E. The annual rainfall is 350 mm per annum, with no rainfall in June and the highest, 55 mm in January. The maximum temperature is 45^o^C, and evaporation rates are 2500 mm per annum. Musina Municipality lies within the catchment of the Limpopo, Nwanedi and Nzhelele rivers, mainly used for irrigation purposes. Musina Local Municipality was selected based on the rampant water-related challenges. The population in the municipality is 32 009 and each household houses an average of five people. The bushveld vegetation contains low lying shrubs and thorny trees such as *Colophospermum mopane* and *Combretum apiculatum*. Main agricultural activities that are conducted in the study site are horticulture using irrigation from Nwanedi and Limpopo River, wildlife management and livestock production. Cattle takes up 27% of the total number of livestock produced in the municipality.

### Research design, sampling procedure and data collection

A sequential mixed method approach was used in the 3-phase study where qualitative methods were followed by a quantitative method. The use of two phases of qualitative methods allowed similarities in experiences to be captured to ensure consistency and accuracy of the control strategies. They catered for variances in context and captured the perceptions, feelings, skills and experiences of indigenous knowledge practitioners. The qualitative phases informed the designing of the data collection tool for the quantitative phase. Triangulation of data collection tools, methods and research participants allowed a comprehensive understanding of IK strategies for ensuring water security of cattle. The study was conducted during December when ambient temperatures reached 42^o^C. The rain had been delayed for three months, at the time of data collection.

#### Face-to-face interviews

Face-to-face interviews were used to gather information on the IK of controlling GIN. The IK included techniques, skills and experiences in managing GIN infestations. The interviews captured details of control measures employed to curb GIN in a drought-prone environment. Each interview generated insights on the importance of controlling GIN, the impact of GIN infestations during droughts and the control strategies used. Eight experts of IK were interviewed comprising of six men and two women aged between 38 and 80 years, as shown in Table 1. The interviews were transcribed and translated into English.

#### Group discussion

The experts who participated in the first phase were grouped for a discussion to rank the strategies for controlling GIN. In the group discussion, the eight experts ranked the methods according to their contribution towards controlling GIN. The ranked methods were diagnoses, treatment and susceptibility to GIN. They further assisted in designing the third phase to support their assertions on the control strategies used when managing GIN in cattle. The focus-group discussion took approximately 90 min and was recorded using a tape recorder and note-taking. The ranking was conducted using a participatory ranking methodology.

#### Non-participant observations of cattle

Led by the IK experts, susceptibility of cows to GIN infestation were demonstrated to reinforce the interviews and the focus group discussion methods. Non-participant observations were used to determine the classes of cattle that were susceptible to GIN. Systematic random sampling was used to select households which participated in the study. The herd for every second household was selected for observation. The owner of the selected herd should have been practising IK to manage his/her cattle. If the owner used conventional methods or integrated them with IK, his or her herd would not be included in the study. Among each selected herd, cattle were sampled based on the following criteria: production system (free-range), genotype (non-descript), age (> 6 months) and the cattle defecated while the research group was present.

**Table 1:** Description of experts of indigenous knowledge

Expert	Gender	Age	IK specialty
1	Male	63	Traditional leader
2	Male	80	Cattle health expert and farmer
3	Male	67	Traditional leader and cattle farmer
4	Female	51	Traditional healer
5	Male	38	Cattle farmer
6	Male	49	Cattle farmer
7	Female	57	Livestock health expert and farmer
8	Male	65	Cattle farmer

Calves below six months were penned and relied solely on milk. The cattle had large flight zones, which made handling for the collection of faecal samples through rectal palpation difficult. The experts ranked the GIN burdens as low, moderate and high. A total of 86 cattle were sampled, which consisted of 15 calves, 25 yearlings and 46 cows. No bulls were part of the sampled animals.

A record sheet was used to record the body condition, class, sex, coat colour, pregnancy and lactation status (as directed by the outcome of the interviews) with the assistance of the owner and through observations. The experts hypothesized that the predisposing factors considered in IK were important in identifying cattle susceptible to high worm loads during droughts.

### Data and statistical analyses

The data from the interviews was transcribed and translated into English. Statistical analyses of cattle observations were conducted using SAS version 9.4 (2013). The association between predisposing factors (body condition, class, sex, coat colour, pregnancy status and lactation status) and GIN burdens were determined using chi-square tests. Ordinal logistic regression was used to estimate the probability of cattle having high GIN burdens. The logit model of PROC LOGISTIC in SAS 9.4 (2013) was used to fit the predictors suggested by experts. An alpha error of 0.05 was used. The logit model used was:

$$In \left[\frac{p}{\left(1-p\right)}\right]={{\upbeta }}_{0}+{{\upbeta }}_{1 }{\text{X}}_{1}+{{\upbeta }}_{2} {X}_{2}\dots +{{\upbeta }}_{t}{\text{X}}_{t}+{\upepsilon }$$

**where;**

P = probability of cattle having high GIN burdens;

[P/1 − P] = odds ratio (the odds of cattle having high GIN burdens);

β_0_ = intercept;

β_1_ × _1_ …β_t_X_t_ = regression coefficients of predictors;

ε = random residual error.

## Results

### Effects of GIN infestation and importance of controlling nematodes in cows

Face-to face interviews revealed that GIN could not be eradicated from cattle. It was essential to manage the infestations so that the burdens remained low. Seasonal and prolonged droughts were frequent, and the heavily-infested cattle were severely impacted, resulting in mortality. Cattle that got into droughts with high worm loads were at risk of deteriorating faster. High burdens of GIN reduced productivity.

Heavily-infested cows had low fertility, aborted and produced less milk. Heavy infestation reduced the growth and chances of survival of the calves. The calves, further, reached sexual maturity much later than calves with low burdens, resulting in the age of first calving being more than two years for heifers.

Heavily-infested bulls became emaciated, and their fertility was reduced. Meat from GIN-infested cattle was of poor quality. Consumption of offals from infested cattle increased the risk of transmission of GIN to humans. Droughts required all classes of cattle to be in condition so that they would fend for themselves. The cattle travelled long distances to access feed and water, but heavy infestation impeded the ability to free-range resulting in general weakness and mortality. High worm loads increased pressure on the farmers to nurse the heavily infested cattle. They had to buy medication, supplementary feed and provide drinking water to ensure the survival of the cattle during droughts. It was, therefore, necessary for the GIN burdens to be controlled as this affected both the farmers and the cattle themselves.

### Indigenous methods of GIN control in cows

Gastrointestinal nematodes control was ongoing and included diagnosis, treatment of GIN infestations and identifying susceptible cattle.

#### Indigenous methods of diagnosing GIN in cattle

Dung consistency and appearance were indicators of GIN burden (Table 2). A mixture of loose dung and solid pellet-like parts, mimicking that of goats, was associated with low-level GIN burdens. Loose dung, which soiled the hind legs, indicated moderate infestation levels with adult worms. Loose dung with transparent, plastic-like covering (mucous) found either.

**Table 2:** Indigenous methods for diagnosis and control of gastrointestinal nematode burdens in cattle

Indicator	Level of infestation	Intervention
Normal dung	None	-
Dung has a mixed consistency	Low	Check the performance of the herd closely.
Dung is of loose consistency, which can soil the hind legs	Moderate	Administering medicinal plants
‘Plastics’ intricately embedded into the faecal heap	High levels of adult worms	Isolation of sick cattle; combining medicinal plants to treat until signs of high worm loads disappear.

embedded inside the dung or outside indicated high worm burdens. Diagnosis of GIN influenced the treatment method employed.

#### Indigenous methods of treating GIN infestations

The experts indicated that they did not treat their cattle for GIN unless they came across faecal heaps showing moderate or high worm burdens. The herds were inspected daily for intestinal worms. If faecal inspections showed that the worm burdens were low, it indicated that the herd was to be monitored closely. Moderate worm burdens were an indication that medicinal plants were to be administered.

The medicinal plants administered included Aloe (*Tshikopa* in Tshivenda) leaf crushed and mixed with water or ground *Mtutula* roots mixed with water and/or ground leadwood (*Combretum imberbe* or *Mudzwiri* in Tshivenda) roots mixed with water. High levels of worm burdens called for the isolation of infested cattle and the continued administration of different medicinal plants combined. The experts did not identify the types of intestinal worms affecting their cattle.

Two experts indicated that treating infested cattle required isolation and the provision of medicine to treat the infestation. In some instances, the worms caused a lot of damage such that reversing it would be impossible. The irreversible damage was indicated by blood, watery faecal heaps with plastics and poor body condition. Continued weakness in treated cattle indicated irreversible damage.

#### Indigenous methods of identifying susceptible cows

Susceptible cattle were identified through knowledge of predisposing factors. The knowledge was necessary when buying new stock. Farmers selected resilient cattle over susceptible ones. At the beginning of a severe drought, the knowledge informed destocking decisions to mitigate losses due to droughts.

Farmers who owned cattle that were susceptible to GIN were better equipped to prepare for droughts by knowing predisposing factors. They stocked up hay or lucerne in preparation for a drought. During droughts, weak cattle that could not withstand travelling long distances, camped along river/stream banks where they defaecated. Because the level of water was low, the worms were not washed away fast enough hence they re-infected the cattle. Many cattle from different villages congregated at these water points causing mass infestations with GIN. Since cattle cannot survive without water, it was important for cattle to be resilient to GIN infestations. There was a consensus that creating a herd that can adapt to harsh environments was important.

*‘…tolerance to harsh environments is important when selecting cattle to include in our herds. That is the reason why our Tshivenda cattle today are more resistant to the nematodes as compared to cattle that came from other areas*.’- Expert 7, Domboni Village.

### Ranking of indigenous methods of controlling GIN

Identifying susceptible cattle was ranked highly, followed by diagnosis and then treatment. Identifying susceptible cattle was necessary due to the notion that ‘prevention is better than cure’. While infestation by GIN could not be avoided in grass-based systems, susceptibility to their adverse impacts was concerning. Assessing the resilience of cattle allowed susceptible cattle to be singled out. After that, decisions on whether to cull the susceptible cattle before a drought or to prioritise them in resource allocation would be made.

After assessing for susceptibility to GIN, diagnosis and treatment were conducted on the cattle. Diagnosis was the second most crucial method in controlling GIN. Diagnosis was described as detecting cattle suffering from the effects of GIN infestations. Positive cattle were those that were failing to fight off the GIN in their system, which was common during droughts. Incorrect diagnosis resulted in either ineffectiveness in the treatment regime due to poor timing or using the wrong treatment regime.

### Predisposing factors of GIN in cows

The experts defined a predisposing factor as one that caused cattle to be susceptible to the harmful effects of high burdens of GIN. During the wet season, cattle could fight off GIN; hence they were primarily asymptomatic. During droughts, however, high GIN burdens caused bleeding. The worms utilised the little feed consumed by the cattle, causing weakness and leading to mortality. The predisposing factors that were identified increased cattle’s susceptibility to high burdens of GIN. The experts ranked the predisposing factors according to their relevance to drought as body condition, class, sex, coat colour, pregnancy status and lactation status (Table 3).

Emaciated cattle had the highest chances of having high GIN burdens. Body condition was the most important factor in determining whether cattle could withstand further stresses caused by droughts. The body condition of cattle was a good indicator of the resilience to GIN. As a result, those cattle that were too fat or thin were susceptible to GIN. Large-bodied cattle were rarely found during droughts; hence thin cattle were appropriate for water-stressed conditions. The cattle with average body size had low burdens of GIN. Thin cattle had visible spine, ribs, pins and poor muscling.

**Table 3:** Predisposing factors of gastrointestinal nematodes in cattle considered in indigenous knowledge

Rank	Predisposing factor		Susceptibility to GIN
1	Body condition	Thin	High
		Satisfactory	Low
		Fat	High
2	Class	Calf	High
		Yearling	Low
		Adult	High
3	Sex	Male	Low
		Female	High
4	Coat colour	Light coloured (Grey, fawn, white)	High
		Dark (Dark brown; Black)	Low
5	Pregnancy status	Pregnant	High
		Non-pregnant	Low
6	Lactation status	Lactating	High
		Non-lactating	Low

Cattle with satisfactory body condition had invisible bony areas. The brisket and a fully muscled brisket and tailhead without fat, was satisfactory. Fat cattle had extremely fatty brisket and tailhead and full muscling.

‘…*we know that if cattle get into a drought thin, they will not make it. The drought will exert too much pressure on the thin cattle through high temperatures and a lack of feed and water. Obese cattle will die from the heat. Now imagine if the cattle have high burdens of worms on top of that, they will not survive. That is why body condition is the most important factor in relation to drought’* – Expert 5, Malale Village.

The class of cattle was a predisposing factor to GIN infestations. Calves were those between the ages of 6 and 12 months and had not been weaned and foraged with the rest of the herds. Yearlings were heifers and steers between the age of 12 and 48 months. Adults were cows and bulls above the age of 48 months. Calves and adult cattle were susceptible to high burdens of GIN. Calves supplemented their milk with solid feed acquired through free-ranging. The calves utilised lower parts of grasses or browsed during droughts. Due to the management strategies employed, adult cattle were susceptible, especially during droughts. The ability to fight off the worms reduced with age of the cattle, especially in cows. Nulliparous and primiparous cows had little problems with GIN burdens compared to those that had calved more than once (multiparous).

All experts were unanimous in identifying sex as a predisposing factor. Cows were more susceptible to GIN as compared to their male counterparts. Among all animal species, males dealt with diseases and parasites better than females. It was the same with cattle in that cows were mainly affected by the GIN compared to bulls. The reason was that cows went through pregnancy and lactation, which weakened their immunity.

Pregnancy and lactation status were also identified as predisposing factors. Pregnant and lactating cows were susceptible to high GIN burdens. During pregnancy and lactation, the cattle focused their energy on sustaining the growing fetuses and calves. Pregnancy and lactation also worked against the long-term immunity of cows. The more the cows calved, the more susceptible they became. In addition to GIN, pregnant cows were affected with dystocia. During droughts, further management and monitoring of pregnant and lactating cows was critical to ensure their reproductive performance.

Stage of production was also a predisposing factor of cows to high GIN burdens. Bulls’ production stage was not considered due to their superiority in curbing GIN burdens. They expended their energy in ensuring the cow’s growth compared to fighting an infestation. This caused them to have higher worm burdens than non-lactating and non-pregnant cows.

Coat colour influenced the susceptibility of cattle to GIN. Light-coloured cattle were more resistant to GIN, which is why farmers preferred fawn and grey-coloured cattle. White, greys and fawn were classified as light coat colours, while black and dark brown was dark. Cattle with mixed coat colours were classified based on the dominant colour. During drought, the light-coloured cattle performed better in the presence of heat and lack of water. When there was a challenge with GIN, therefore, light-coloured cattle resisted the infestations.

### Associations between predisposing factors and GIN burdens

The associations between predisposing factors and GIN burdens are shown in Table 4. There was a significant association (*P* < 0.05) among sex, coat colour, pregnancy status, and GIN burdens. Dark-coloured, pregnant cows were susceptible to high GIN burdens. Table 5 shows the odds ratios of different classes of cattle having high burdens of GIN. Female calves, heifers and cows were 2.6 times more likely to have high worm burdens than male calves and yearlings. Cattle with dark coat colours were 3.5 times more likely to have high GIN burdens than light-coloured ones. The likelihood of pregnant cows having high GIN burdens was 4.9 times higher than the non-pregnant cows.

## Discussion

Indigenous systems have equipped farmers with skills and knowledge to control GIN by identifying susceptible cattle, diagnosis, and treatment. The findings of this study have provided insights into the control of GIN under IK and the predisposing factors that are considered. Evaluating the predisposing factors has gone further to quantify their association with GIN burdens during droughts.

Understanding this perspective is essential as it reflects how cattle can be managed to ensure their survival. Understanding the predisposing factors and their association with GIN burdens will assist in developing cattle management strategies such as selection for drought tolerance when buying new stock or selling and culling (Gillandt et al., 2018). In that regard, IK becomes convenient when preparing, mitigating and adapting to droughts.

Indigenous knowledge system has been used to identify the clinical signs of GIN infestations.

**Table 4:** Association among predisposing factors and GIN burdens of cattle in drought conditions

		Chi-square value	Significance
Body condition	Thin	3.88	NS
	Satisfactory		
Class	Calf	8.75	NS
	Yearling		
	Adult		
Sex	Male	6.18	*
	Female		
Coat colour	Light	9.32	*
	Dark		
Pregnancy status	Pregnant	8.86	*
	Non-pregnant		
Lactation status	Lactating	0.01	NS
	Non-lactating		

NS: not significant (*P* > 0.05); *P < 0.05

**Table 5:** Odds ratio estimates, lower confidence intervals (LCI) and higher confidence intervals (HCI) to predict the GIN burdens of cattle under drought conditions

	Estimate	LCI	HCI	Contribution	Significance
Sex	2.639	0.150	2.954	0.30	*
Coat colour	3.498	1.488	8.228	0.17	*
Pregnancy status	4.878	0.066	5.637	0.14	*
Body condition	1.132	0.036	2.005	0.04	NS
Class	0.982	0.233	1.694	0.03	NS
Lactation status	0.763	0.482	1.231	0.05	NS

^*^P < 0.05. The R-square value for the model was 0.71

The signs identified were consistent with parasitic gastroenteritis (PGE). The clinical signs of PGE are watery diarrhoea, emaciation and poor coat condition (Eysker and Ploeger, 2000). These clinical signs are consistent with infection with *Ostertagia ostertagi*, *Cooperia* spp., *Trichostrongylus* spp and *Haemonchus* spp. Scouring occurs after the intestinal lining has been damaged by the burrowing action of nematodes reducing the absorption of water from the digesta into the body (Underwood et al., 2015). Even though IK could not be used to classify GIN species, scouring is consistent with *Haemonchus* spp. infestation (Underwood et al., 2015). Faecal matter of mixed consistency may show early signs of GIN.

The use of phytotherapy has been reported in other studies. The medicinal plants used in IK may treat a broad spectrum of intestinal parasites (Kundu et al., 2014), thereby shadowing the need to classify and treat the GIN according to species. *Combretum spp*., for example, contains oligomeric proanthocyanidins responsible for treating *Haemonchus contortus* and *Caenorhabditis elegans* infestations (Simon et al., 2012; Spiegler et al., 2015). *Aloe spp*. contains bioactive compounds such as mangiferin, rutin, quercetin, and β-sitosterol, responsible for larval mortality of *H. contortus*, *Trichostrongylus*, *Chabertia* and *Teladorsagia*/*Ostertagia* (Giovanelli et al., 2018; Chitura et al., 2019). Contrasting reports of efficacy in reducing GIN burdens have been reported (Chitura et al., 2019). The different reports concerning efficacy rationalise the idea of communal farmers using combinations of medicinal plants.

Predisposing factors of GIN in the communal setting, which were investigated using the conventional knowledge system, include age (León et al., 2019), sex, treatment status, body condition (Telila et al., 2014; Gunathilaka et al., 2018), season (Dreyer et al., 1999), lactation status (da Silva et al., 2012) and breed (Marufu et al., 2011). Both indigenous and conventional systems consider relatively the same predisposing factors, although their degree of risk may vary from place to place. This study has revealed that IK considers two categories (thin, satisfactory and fat) when evaluating body condition for assessing predisposing factors. Conventional body condition scoring considers a nine-point system to score three conditions fat, medium and lean (Nicholson and Butterworth, 1986) and a five-point system which classifies thin (score 1) to excessively fat (Score 5) (van Niekerk and Louw, 1980). The IK body condition scoring aligns with that of Nicholson and Butterworth (1986). Farmers can capitalise on the knowledge of predisposing factors through education via IK holders within their communities. The observation that farmers did not identify parasitic factors such as species of GIN affecting the cattle was because IK has developed treatments that utilise concoctions of broad-spectrum medicinal plants (Kundu et al., 2014) hence shadowing the need to know the types of GIN.

The study suggests that the influence of stressors associated with droughts in communal production systems exposes all cattle, regardless of body condition, class or lactation, to opportunistic infections, including GIN. This may imply that when drought conditions intensify, the influence of these factors is uniform. That partly explains why body condition and lactation status were not significant in the prediction of worm burdens. In this regard, the assertions by the experts of IK may be valid. The finding that farmers considered body condition when assessing cattle for susceptibility to GIN infestations was consistent with Gunathilaka et al. (2018). The observation that emaciated cattle have relatively the same risk as satisfactory is unexpected. As body condition increases, cattle expend reduced amounts of worm loads (Telila et al., 2014). In the conventional knowledge system, there are still mixed conclusions among scholars concerning the influence of age/class on the risk of gastrointestinal parasitism. León et al. (2019) did not find an association between class and gastrointestinal parasitism, while Gunathilaka et al. (2018) reported an association with the calves being more susceptible than adult cows. The infestation by *Spongyle spp*. is the same throughout all classes (Telila et al., 2014).

Light-coloured cattle have higher resilience to droughts due to their ability to resist hydric and thermal stress by reflecting heat compared to dark ones, which absorb heat (Brown-Brandl, 2018; Anzures-Olvera et al., 2019). In addition, Tőzsér et al. (2003) revealed that black cattle show a high temperament, agitation and discomfort than lighter-coloured ones in the face of hydric and thermal stressors. Cattle with high temperaments exhibit high-stress levels and inadequate immune responses (Burdick et al., 2011). It can, therefore, be expected that light-coloured cattle perform better when additional stress due to GIN infestations is exerted.

The high susceptibility of females compared to males has contrasted with the findings in Sri Lanka, where the males were more susceptible (Gunathilaka et al., 2018). The difference between Gunathilaka et al. (2018) and the current study was the influence of a water-stressed environment. The higher susceptibility in cows may be due to the influence of pregnancy, parturition and lactation, which reduce their immunity (Elsheikha, 2017). The high susceptibility of pregnant cows to GIN may be attributed to the peripartum immunological relaxation (da Silva et al., 2012).

Cattle observations isolated sex, coat colour and pregnancy status as the noteworthy predisposing factors in drought conditions. Sex and coat colour, as genetic factors, may be used to select resilient foundation stock as a preventative and/or adaptive strategy. Foundation stock may include more male cattle to buffer the herds against losses due to droughts. Farmers may also include light-coloured cattle such as the Tuli, characterised by light-coloured coats, particularly red, yellow and white. The multicoloured Nguni should have light primary colours (Nyamushamba et al., 2017).

Pregnant cows had the highest risk of GIN burdens. The findings reinforce the recommendations that pregnant cows should be culled first in a destocking programme for drought mitigation (Gill and Panchak, 1999). In communal settings where off-take rates are low due to owners’ attachment to their cattle (Mmbengwa et al., 2015), these findings strengthen the need to prioritise pregnant cattle due to their susceptibility to GIN and the subsequent impacts of drought. After farmers receive a warning of impending drought, they may stock up on feed and work towards providing water to pregnant cows. This may be done so that the cows do not rely on free range during the drought. A reduction in the immune response can be experienced under high temperatures and water scarcity (Ndou et al., 2011). Physical strain and thermo-hydric stress on pregnant cows, therefore, should be minimised to strengthen their immunity against GIN. This may be achieved by constructing shades in the rangelands and reducing the distance to water sources.

## Conclusions

Indigenous knowledge of controlling nematodes in Southern Africa involves identifying susceptible cattle, diagnoses and treatment of infestations. The utilization of IK, therefore, extends beyond ethnoveterinary medicine and the treatment of symptoms of parasites. Diagnoses assesses faecal appearance and treatment utilises phytotherapy. The most important method was identifying susceptible cattle. A dark-coloured, pregnant cow is highly susceptible to high GIN burdens during droughts. Susceptible cows are then excluded when buying foundation stock or prioritised in the allocation of feed and water during droughts. Where resources were unavailable, they are culled to mitigate losses due to droughts. Gastrointestinal nematodes negatively affect reproduction of cows, hence in addition to controlling GIN, strategies of sustaining reproductive efficiency should be investigated.

## Data Availability

Data will be made available upon request.
